# Genetic polymorphisms and plasma concentrations of leptin (rs7799039) and adiponectin (rs17300539) are associated with obesity in children and adolescents

**DOI:** 10.1590/1984-0462/2022/40/2021030IN

**Published:** 2022-06-10

**Authors:** Carlos Alberto Menezes, Eduardo Rodrigues Alves, Gustavo Nunes de Oliveira Costa, Thaís Caroline Dallabona Dombroski, Rafael Teixeira de Mattos, Juliana de Assis Silva Gomes, Fabricio Rios-Santos

**Affiliations:** a Universidade Estadual de Santa Cruz, Ilhéus, BA, Brazil.; b Centro Universitário de Várzea Grande, Várzea Grande, MT, Brasil.; c Universidade Salvador, Salvador, BA, Brazil.; d Universidade Federal de Minas Gerais, Belo Horizonte, MG, Brazil.; e Universidade Federal de Mato Grosso, Cuiabá, MT, Brazil.

**Keywords:** Childhood obesity, Genes, Biomarkers, Obesidade infantil, Genes, Biomarcadores

## Abstract

**Objective:**

The aim of this study was to compare the anthropometric, biochemical, and hormonal characteristics and the presence of genetic polymorphisms of leptin, adiponectin, and tumor necrosis factor alpha (TNF-α) between eutrophic and obese children and adolescents.

**Methods:**

This is a case–control study involving 104 children and adolescents. All subjects were assessed for anthropometric characteristics and clinical, laboratory, and genetic polymorphism parameters. The sample was selected from the pediatric endocrinology outpatient clinic specialized in the treatment of obesity in children and adolescents according to the Centers for Disease Control and Prevention (CDC) classification, and controls were selected from the same location in the general pediatric outpatient clinic.

**Results:**

As a result, the parameters, such as black color, obese parents, hypertensive parents, and early weaning, were found to be associated with obesity. Increased levels of insulin, triglyceride, total cholesterol, LDL cholesterol, CRP-U, AST, ALT, GGT, free T4, IGF-1, and uric acid and low levels of HDL cholesterol are found to be associated with a higher chance of obesity. The presence of AG/AA polymorphisms in the leptin is associated with a 290% (OR 3.9) higher chance of obesity, and for adiponectin genes, the chances are 740% (OR 8.4) higher. In these obese children and adolescents with AG/AA haplotypes, serum leptin levels were increased and adiponectin levels were decreased in eutrophic individuals, whereas serum TNF-α levels did not change.

**Conclusions:**

The AG/AA polymorphisms in the leptin and adiponectin genes alter the serum levels of these adipokines and predispose them to obesity, and many anthropometric, biochemical, and hormonal markers are altered, demonstrating early consequences for the health of these obese children and adolescents.

## RESUMO

**Objetivo:**

Comparar as características antropométricas, bioquímicas, hormonais e a presença de polimorfismos genéticos de leptina, adiponectina e fator de necrose tumoral alfa (TNF-α) entre crianças e adolescentes eutróficos e obesos.

**Métodos:**

Trata-se de um estudo caso-controle conduzido com 104 crianças e adolescentes. Todos os indivíduos foram avaliados quanto às características antropométricas e parâmetros clínicos, laboratoriais e de polimorfismo genético. A amostra foi selecionada no ambulatório de endocrinologia pediátrica especializado no tratamento da obesidade em crianças e adolescentes de acordo com a classificação do Centers for Disease Control and Prevention (CDC), e os controles foram selecionados no mesmo local, porém no ambulatório de pediatria geral.

**Resultados:**

Alguns parâmetros foram associados à obesidade em nosso estudo: cor preta, pais obesos, pais hipertensos e desmame precoce. Níveis aumentados de insulina, triglicerídeos, colesterol total, colesterol LDL, PCR-U, AST, ALT, GGT, T4 Livre, IGF-1, ácido úrico e níveis baixos de colesterol HDL estão associados a uma chance maior de obesidade. A presença de polimorfismos AG/AA na leptina está associada a uma chance 290% (OR 3,9) maior de obesidade, enquanto para os genes da adiponectina as chances são 740% (OR 8,4) maiores. Nessas crianças e adolescentes obesos com haplótipos AG/AA, os níveis séricos de leptina aumentaram e os níveis de adiponectina diminuíram em relação aos eutróficos, já os níveis séricos de TNF-α não se alteraram.

**Conclusões:**

Concluiu-se que os polimorfismos AG/AA nos genes da leptina e adiponectina alteram os níveis séricos dessas adipocinas e predispõem à obesidade precoce, e muitos marcadores antropométricos, bioquímicos e hormonais ficam alterados, trazendo consequências para a saúde dessas crianças e adolescentes.

## INTRODUCTION

According to the World Health Organization (WHO), there was an estimated 1.9 billion (39%) overweight and obese adults. Overweight children and adolescents between the ages of 5 and 19 years are 340 million. By 2025, it is expected that there will be an increase in the overweight people by 2.3 billion.^1,2^

Obesity is determined by a combination of genetics, environment, and lifestyle. It is known that obesity initiates during childhood or adolescence, followed by increased rates of dyslipidemia, hypertension, coronary diseases, diabetes, and depression before adult age.^1,3^

Adipose tissue and regulation of leptin, adiponectin, and tumor necrosis factor alpha (TNF-α) are linked to endocrine and molecular mechanisms, which play a physiological role in energy balance and body weight. In this sense, the human leptin (*LEP*) gene has the function of informing the brain about energy supply, in addition to the modulation of glucose and lipid metabolism, angiogenesis, immunity, and homeostasis of blood pressure. Serum leptin production and concentration are proportional to adipose tissue mass.^4–7^

In contrast, the adiponectin gene produces a protein exclusively transcribed in adipose tissue. It reduces TNF-α expression, decreases macrophage chemotaxis, inhibits monocyte adhesion and macrophage transformation into foam cells, increases nitric oxide production, and stimulates angiogenesis. Due to its multiplicity of beneficial functions, adiponectin is the only adipokine with anti-inflammatory, antidiabetic, and antiatherogenic effects. The *TNF-*α gene, in turn, is associated with obesity and insulin resistance, and hyperandrogegism.^5,8,9^

The presence of polymorphisms in the leptin, adiponectin, and TNF-α genes in adult individuals may alter serum adipokine concentrations and predispose obesity. Therefore, it is necessary to investigate this relationship in children and adolescents with these polymorphisms and measure the consequences on body weight and health.^5–7^

Therefore, the objective of this study was to compare anthropometric, biochemical, and hormonal parameters and the presence of genetic polymorphisms of leptin, adiponectin, and TNF-α among eutrophic and obese children and adolescents.

## METHOD

This is a case–control study involving 104 individuals (58 obese and 46 eutrophic) aged 6–18 years, with a mean age of 10±2 years. The subjects were obese patients who visited at the Preventive Medicine Service, Aracaju, Sergipe, Brazil, in the period May–November 2019. The patients were divided into case group and control group. The case group included those who had body mass index (BMI) above the 97th percentile or Z-score greater than +2, according to the Centers for Disease Control and Prevention (CDC) classification. The patients were excluded if they have taken anti-obesity or anti-anxiolytic medication and those with bulimia. The control group consisted of patients selected from the same location in the general pediatric clinic, who considered themselves healthy, were eutrophic with a BMI of the 50th percentile or a Z-score of 0, and age similar to the case group.^10^

All subjects were evaluated for anthropometric characteristics, sex color, obese father, obese mother, hypertensive father, hypertensive mother, type of birth, early weaning, and food behavior; and in clinical parameters, birth weight, BMI, and abdominal circumference were assessed.

The laboratory evaluation was performed in a certified laboratory from the serum sample taken after 12 h of fasting. The sample included concentration of 21 biochemical, hormonal, and cytokine parameters, such as insulin, glucose, homeostatic model assessment for insulin resistance (HOMA-IR), triglyceride (TRI), total cholesterol, high-density lipoprotein (HDL), low-density lipoprotein (LDL), ultrasensitive C-reactive protein (CRP-U), homocysteine, interleukin 10 (IL-10), IL-6, aspartate aminotransferase (AST), alanine aminotransferase (ALT), gamma-glutamyl transferase (GGT), thyroid-stimulating hormone (TSH), free T4 (T4L), insulin-like growth factor 1 (IGF-1), cortisol, uric acid, leptin, adiponectin, and tumor necrosis factor alpha (TNF-α).

Single-nucleotide polymorphisms (SNPs) were selected using the SNP browser TM software version 4.0 (www.allsnps.com). This program illustrates a panel of human chromosomes representing the location of more than 150,000 SNPs validated in the haplotypic blocks and respective SNP tags for each population evaluated in the HapMap Project (International HapMap Consortium, 2005), in addition to prioritizing the selection of SNPs based on the model’s connection imbalance.

The criteria used for the selection of the markers were the presence of the marker in the four HapMap phase II populations, the frequency of the smallest allele (MAF — minor allele frequency) greater than 5%, and the presence of at least one marker in each haplotypic block of the genes. Therefore, we studied the SNPs and compared the association of genetic polymorphisms of leptin (rs7799039, 2548G/A), adiponectin (rs17300539, 11391G/A), and TNF-α (rs1800629, 308G/A).

After extraction of the genomic DNA by the Flexi Gene® kit (Qiagen), SNP analyses on the adiponectin rs17300539 (11931G/A) and leptin rs7799039 (2548G/A) genes were performed using the real-time polymerase chain reaction (PCR). From the isolated DNA, the DNA fragments were amplified by the commercial TaqMan system, using primers and probes corresponding to each SNP made by Applied Biosystems®.

The specific primers used for the genes in question are as follows: SNP rs7799039 (leptin) (5’-TTGTTTTGTTTTGCGACAGGGTTGC [A/G] CTGATCCTCCCGCCTCAGTCTCCCT-3’) and SNP rs17300539 (adiponectagA) [TipGTTAA] GCTCAGATCCTGCCCTTCAAAAACA-3’).

Reactions were performed in CFX96 real-time thermal cycler (Bio-Rad®). A final reaction volume of 20 μL containing 50 ng of DNA diluted in water, 10 μL of 2× master mix (TaqMan), and 1 pmol/μL of primer were used. The analyses were performed using the CFX 96 (Bio-Rad®) thermocycler program.

To identify the SNP in the TNF-α rs1800629 (−308G/A) gene, PCR-RFLP was performed using specific primers to amplify the polymorphism region, with primer forward (5’’AGGCAATAGGTTTTGAGGGCCAT-3’) and reverse (5’’TCCTCCCTGCTCCGATTCCG-3’) as previously described.^11^

The enzymatic restriction reaction with the Ncol enzyme was standardized at 37°C for 2 h. The electrophoretic run was performed on a 2% agarose gel at 125 V for 1 h and 20 min to analyze the products of the PCR-RFLP reaction with Syber green intercalating dye.

The bands were visualized by an image capture system coupled to a transilluminator. A 107 bp band, without the restriction of the Ncol enzyme, corresponds to the wild-type homozygote genotype (GG), and the presence of two 87 and 20 bp bands corresponds to the cut by the enzyme, due to the presence of the allele (A), polymorphic homozygote (AA), and the presence of three bands, 107, 87, and 20 bp, corresponding to the heterozygote (GA).

The analyses were performed using STATA Analysis and Statistical software version 12 (StataCorp LLC, Texas, USA).

To evaluate the normality of the laboratory parameters, a graph of diagnosis for quantile–quantile (Q-Q) residues was used in a regression model, and the significance value (p) of the Shapiro–Wilk test for the diagnosis of normality was considered as a deciding factor.

The nonparametric study variables were presented as frequency and median and dispersion measures as the interquartile range (25th percentile–75th percentile).

To compare the quantitative results between the eutrophic and obese classification, the data were compared with the Mann–Whitney U test, considering the level of significance (α) as 5% (0.05).

To compare the qualitative results between the eutrophic and obese classification, homogeneity tests were performed using the chi-square test and *Odds Ratio* (OR) statistics with 95% confidence intervals (95% CI) to estimate the associated risk.

In this study, 60 patients met the inclusion criteria, and 58 agreed to participate in the study. Among the 60 (eutrophic), 14 were dropped out during the survey. Considering a 95% CI, the cases have a sampling error of 2.3% and the controls 7.0%.

The ethical and methodological aspects of this study were approved by the local Ethics Committee (protocol 04065412.600005526) according to the National Health Council (Resolutions 466/12). All participants were informed about the objectives and procedures of the study, and written informed consent was obtained from the participants.

## RESULTS

The eutrophic group was composed of 46 children and adolescents with a mean age (SD) of 10 (±2.7) years and had a BMI with a mean (SD) of 17.3 (±2.1), and the obese group was composed of 58 children and adolescents with age mean (SD) of 11 (±2.9) years and presented BMI with mean (SD) 28.1 (±5.3).

Anthropometric characteristics were compared between eutrophic and obese children and adolescents. The parameters such as skin color (black), having an obese father or mother, having a hypertensive father, and having been weaned early, that is, before 6 months of life were found to be associated with obesity (Table 1).

**Table 1:** Relative frequency and comparison of anthropometric characteristics of 104 eutrophic and obese children and adolescents treated at the Preventive Medicine Service in the city of Aracaju-SE, Brazil.

Characteristics		% classification		*Odds Ratio*	95% CI	p-value^a^
		Obese	Eutrophic			
Sex	Masculine	57	54	1.1	0.5–2.6	0.794
	Feminine	43	46			
Declared color	Black	86	45	7.4	2.2–31.6	<0.001
	White	14	55			
Obese father	Yes	75	39	4.6	1.8–11.8	<0.001
	Não	25	61			
Obese mother	Yes	80	36	7.2	2.7–20.2	<0.001
	No	20	64			
Hypertensive father	Yes	84	49	5.5	1.4–30.9	0.005
	No	16	51			
Hypertensive mother	Yes	68	51	2.0	0.7–5.7	0.132
	No	32	49			
Type of birth	Cesarean	62	42	2.2	0.8–5.7	0.064
	Normal	38	58			
Early weaning(<6 months)	Yes	94	23	49.6	12.2–274.7	<0.001
	No	6	77			

a Chi-square test with *Odds Ratio* and 95% confidence interval (95% CI)

b No case for the control variable.

The HOMA-IR index values in the eutrophic and obese groups were 1.6 and 2.6, respectively (p<0.001), with birth weights of 3,000 and 3,285 kg (p=0.010) and abdominal circumferences of 65 and 88 cm (p<0.001), and these clinical and laboratory parameters were associated with higher statistical significance in the group of children and adolescents with obesity when compared to eutrophic individuals.

The levels of insulin, TRIs, total cholesterol, LDL, CRP-U, AST, ALT, GGT, and uric acid were elevated and HDL levels were decreased among obese patients when compared with eutrophic patients. Although the levels of T4L, IGF-1, and cortisol are high in the obese group compared with controls, these parameters do not meet the criteria of hormonal pathologies that are performed for diagnostic screening (Table 2).

**Table 2:** Comparison between the results of the insulin resistance parameters of 104 children and adolescents selected at the Preventive Medicine Service in the city of Aracaju-SE, Brazil.

Parameter	Control case	P50	P25–P75	p-value^a^
Insulin (mU/L)	Obese	11.3	9–15	<0.001
	Eutrophic	7.2	5.6–9.2	
Glucose (mg/dL)	Obese	89.0	85–93	0.057
	Eutrophic	88.0	80–91	
Triglyceride (mg/dL)	Obese	152.0	89–178	<0.001
	Eutrophic	76.5	66–89	
Cholesterol (mg/dL)	Obese	160.0	139–180	0.001
	Eutrophic	145.0	127–156	
HDL (mg/dL)	Obese	39.0	34–45	<0.001
	Eutrophic	48.0	43–54	
LDL (mg/dL)	Obese	100.0	87–140	0.021
	Eutrophic	89.0	78–101	
CRP-U (mg/dL)	Obese	1.5	0.3–2.03	<0.001
	Eutrophic	0.02	0.01–0.1	
Homocysteine (μmol/L)	Obese	6.0	4.8–7.1	0.144
	Eutrophic	5.4	4.1–7	
IL-10 (pg/mL)	Obese	4.1	3.1–5.3	0.156
	Eutrophic	3.7	3.0–4.4	
IL-6 (pg/mL)	Obese	7.7	7.4–9.8	0.701
	Eutrophic	8.2	7.3–9.5	
AST (U/L)	Obese	23.0	16–32	<0.001
	Eutrophic	12.0	12–17	
ALT (U/L)	Obese	25.0	18–34	<0.001
	Eutrophic	16.0	13–16	
GGT (U/L)	Obese	32.0	22–35	<0.001
	Eutrophic	18.0	15–21	
Thyroid-stimulating TSH (mU/L)	Obese	1.5	1–2.1	0.955
	Eutrophic	1.3	1–2.1	
Free T4 (ng/dL)	Obese	1.0	0.8–1.2	<0.001
	Eutrophic	0.7	0.6–1	
IGF-1 (ng/mL)	Obese	230.0	190–278	<0.001
	Eutrophic	194.0	178–210	
Cortisol (μg/dL)	Obese	7.9	6.5–10.5	0.052
	Eutrophic	9.0	7.3–11	
Uric acid (mg/dL)	Obese	4.3	3.8–5.2	<0.001
	Eutrophic	3.2	2.5–3.8	

a Mann–Whitney U test; HDL: high-density lipoprotein; LDL: low-density lipoprotein; CRP-U: C-reactive protein ultrasensitive; AST: aspartate aminotransferase; ALT: alanine aminotransferase; GGT: gamma-glutamyl transferase; IGF-1: insulin-like growth factor 1.

A greater chance of obesity was observed in patients who had leptin AA/AG haplotypes (rs7799039) (OR 3.9; 95% CI 1.4–12.1; p=0.003) and adiponectin (rs17300539) (OR 8.4; 95% CI 1.8–72.2; p=0.001) when compared to GG haplotypes, but not significant for patients’ TNF-α haplotypes (Table 3).

**Table 3:** Frequency and comparison of adiponectin, leptin, and TNF-α haplotypes among adolescents and obese patients treated at the Preventive Medicine Service in the city of Aracaju-SE, Brazil.

Gene	Haplotypes (n)	Classification (n)		Odds Ratio	95% CI	p-value^a^
		Obese	Eutrophic			
Leptin (rs7799039)	AG/AA (31)	24	7	3.9	1.4–12.1	0.003
	GG^b^ (73	34	39	0.3	0.1–0.7	
Adiponectin (rs17300539)	AG/AA (18)	16	2	8.4	1.8–78.2	0.001
	GG^b^ (86)	42	44	0.1	0.1–0.6	
TNF-α (rs1800629)	AG/AA (29)	18	11	1.4	0.6–3.8	0.421
	GG^b^ (75)	40	35	0.7	0.3–1.8	

a Chi-square test with *Odds Ratio* and 95% confidence interval (95% CI)

b Wild type.

**Figure 1:**
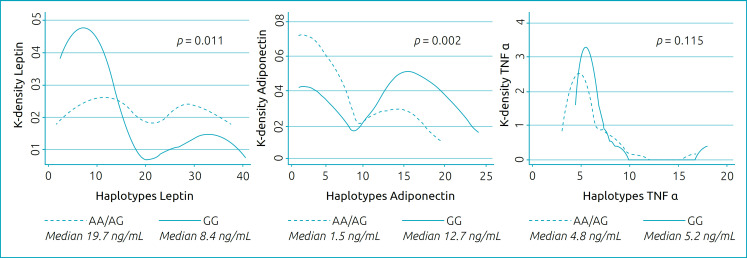
Distribution by density (kernel) of the serum concentration of leptin, adiponectin, and TNF-α in the AA/AG and GG haplotype categories with comparison by Mann–Whitney and presentation of the medians by haplotypes.

Patients with leptin AA/AG haplotypes had higher serum leptin concentrations when compared to the GG haplotype, whereas patients with AA/AG adiponectin haplotype had lower serum adiponectin concentration when compared to the GG haplotype. No difference was found in serum TNF-α concentration between the TNF-α haplotypes of the study patients (Figure 1).

The Kernel density graph shows a predominance of low results for leptin in patients with GG haplotypes, while the adiponectin graph shows a predominance of high results for GG haplotypes (Figure 1).

## DISCUSSION

Several etiological causes may contribute to the onset of children and adolescents’ obesity. The obese children are at higher risk of becoming obese adults and of suffering more serious complications of illness in the short term.^1,2^

In our study, we found higher serum leptin levels in patients with AA/AG haplotype of this gene, which is similar with previous studies in adults. Jiménez-Osorio et al. demonstrated that the presence of AA/AG polymorphism in the leptin gene is associated with obesity in adult patients.^21^ In our sample of children and adolescents, we also found this association. Studies demonstrate that the allele A is a protective factor against obesity in adults.^5,6^ In our study, we can evidence this protection in eutrophic children and adolescents. The relationship between these genetic factors of leptin in the regulation of leptin synthesis is evident, which may be important for therapeutic interventions in obese children and adolescents.

The polymorphism in the leptin gene rs7799039 is located at the 5’ end of the promoter region and close to a transcription factor binding site that regulates leptin transcription. Leptin is an adipokine that regulates food intake by suppressing appetite, and increased serum levels are associated with inflammation, oxidative stress, and the development of insulin resistance.^4,5,20^

The rs17300539 adiponectin gene, an adipokine abundantly produced by adipose tissue, was associated with a decrease in the serum expression of adiponectin in adults.^7,22^ In our study, this decrease was also observed in obese children and adolescents. Adiponectin participates in energy homeostasis and acts on adipose tissue, and reduction in adiponectin concentrations promotes sluggish lipid and carbohydrate metabolism, thereby stimulating the consumption of food for energy supply. The allele A of this gene appears to increase adiponectin levels due to increased transcription, suggesting an influence of this allele on characteristics related to obesity.^23,24^

The role of the environment cannot be ignored, although the genetic variants in the adiponectin and leptin genes have effects on energy consumption and weight gain.^24,25^ However, the understanding of the genetic mechanisms involved in the genesis of obesity is necessary for the development of interventions even in childhood and adolescence that can prevent future health problems, which in some cases are irreversible, but which can be mitigated by a team of interdisciplinary health professionals.

Furthermore, it has been reported that obesity has a hereditary factor and that black adults are more likely to be obese.^3^ This conformed to the children and adolescents in our study. However, our result differs from a study published in 2016 with 885 elderly Brazilians in which obesity was not associated with self-declared black color, but with socioeconomic variables such as education and family income, which may be related to color.^3,12^

Our study demonstrated that babies who started solid feeding before 6 months of age, characterized by early weaning, had a higher chance of obesity in childhood and adolescence, which corroborates with the WHO recommendations of breastfeeding for 6 months, followed by the gradual introduction of nutritionally adequate and safe complementary foods along with the maintenance of breastfeeding for up to 2 years. Other epidemiological studies have also shown that babies who started feeding solids before the age of 6 months have a higher risk of obesity compared to those who started after.^1,2^

In our study, we observed a higher birth weight in obese patients. The fetus reflects, depending on nutrition, growth, and body composition, the supplies and energy it receives from the mother and also expresses its dependence on the placental function.^2,5^ It also receives a flow of chemical mediators, which inform about the mother’s nutritional status and possibly the quality of the postnatal environment. This information contributes to shaping the composition and size of the body, as well as to the structuring of the individual’s endocrine and metabolic systems.^2,4^

Obesity is accompanied by an increase in waist circumference, which was also observed in this present study. Higher waist circumference represents a higher risk for the development of diabetes, dyslipidemia, cardiovascular disease, and metabolic syndrome. This measure has been suggested to be better than BMI to estimate these risks.^2^

Assessing the relationship between diabetes and obesity, in our study, it is possible to observe that in childhood and adolescence, β-pancreatic cells increase their production of insulin as a compensatory mechanism, while glucose tolerance remains normal.^13^

Insulin resistance in obese patients can be explained due to the inflammatory response with alteration of substances that act systemically, participating in several metabolic processes, such as leptin, adiponectin, and TNF-α, which play a fundamental role in insulin resistance.^8,13^

In this situation, there is a decrease in the insulin capacity to stimulate the use of glucose by the muscle and fat tissue, causing damage to the suppression of lipolysis, a condition that increases the circulation of free fatty acids and further alters the transport of glucose to the target tissues inhibiting the action of insulin. Insulin resistance leads to increased oxidation of fatty acids, providing a substrate for the synthesis of TRIs and increasing the release of LDL cholesterol by decreasing HDL cholesterol.^9^

In our study, we identified the change in the levels of the components of the lipid profile, which is explained by the accumulation of adipose tissue and the release of free fatty acids, which are in greater proportion directed to the liver for greater production of TRIs and LDLs, contributing to the increase in total cholesterol and decrease in HDL cholesterol.^14^ This lipid profile is related to an increased risk of developing cardiovascular disease, hepatic steatosis, and insulin resistance.^8,13^

The PCR-U parameter has already been associated with obesity in adults. In our study, PCR-U increase was associated with obese children and adolescents. PCR-U has been described as an important marker of future coronary disorders. The normal levels of a population are below 0.5 mg/dL. The eutrophics in this present study had a median of 0.02 mg/dL, while the obese group had 1.52 mg/dL. Studies show that a small increase is considered a cardiovascular risk factor, independent of other factors already known.^15^ Although PCR-U is not specific for cardiovascular risk, compared to the control group, the highest risk of coronary disorders associated with obese children and adolescents is evident.

The liver parameters such as AST, ALT, and GGT were elevated and compared between obese patients and eutrophic patients, despite not being above the reference value. Obesity is associated with several changes in the liver resulting from the release of fatty acids from adipose tissue for the production of TRIs in the liver, which leads to liver steatosis and increased liver enzymes and, in some cases, may progress to hepatomegaly, fibrosis, and cirrhosis.^16^

It is known that childhood obesity is multifactorial, with the majority of cases being exogenous in nature; however, it can be associated with other causes, especially when accompanied by identifiable changes in growth monitoring. It has been described that hypothyroidism is not a cause of obesity, just as obesity is not a cause of hypothyroidism.^17^ However, in our study, we found higher T4L levels in obese individuals, and this elevation does not meet the criteria for thyroid hormone pathologies. This fact has not been described in the literature.

It is known that hyperinsulinemia, a characteristic that was observed in our sample, induces a higher production of IGF-1 and lower production of IGFBP-1 (Insulin-like growth factor-binding protein 1) by the liver. IGFBP-1 is an IGF-1 binding protein that inhibits its activity. These insulin-induced changes would result in a greater amount of free IGF-1 capable of exerting negative feedback on the pituitary for GH secretion.^18^ Our obese patients had higher levels of IGF-1 when compared to eutrophic individuals.

Uric acid is an organic compound, produced endogenously in the liver of humans. It acts as a metabolite of purines. It is formed by adenosine, inosine, hypoxanthine, adenine, and guanine. It is considered as one of the main hydrophilic antioxidants in the body. Uric acid inhibits the action of free radicals on organic molecules, such as those that make up the cell membrane and genetic material. In our study, obese patients had higher levels of uric acid, and this chronic increase is associated with the risk of metabolic syndrome; however, the acute increase in its concentration seems to be a protective factor against oxidative stress.^19^

Although the sample is considered small for polymorphism studies in chronic metabolic diseases, the population of our study was a multiracial heterogeneous group of children and adolescents with obesity in the Northeast region of Brazil. Further studies should be carried out with children and adolescents with obesity in other regions of Brazil with a larger sample.

We conclude that the AG/AA polymorphisms in the leptin and adiponectin genes alter the serum levels of these adipokines and predispose to obesity. Furthermore, there are changes in anthropometric, biochemical, and hormonal markers, showing early consequences for the health of these children and adolescents.
